# Bassorin Paste

**Published:** 1891-04-18

**Authors:** 


### THERAPEUTICS.

### BASSORIN PASTE.

This is proposed as a substitute for the gelatine base recommended by Unna and others, which, though an extremely useful medium for dermatological remedies, has some disadvantages. Dr. Elliott, of New York, in his contribution to the "Journal of Cutaneous and Genito-Urinary Diseases" for February, 1891, informs us that this paste, which was prepared under his direction by Mr. Lascar, the pharmacist of Demilt Dispensary, is composed chiefly of bassorin, a substance which is defined in Dr. Foster's Medical Dictionary as belonging to the class of vegetable mucilages derived from Bassora gum, tragacanth, and other sorts of gums; tasteless, odourless, and almost colourless; insoluble in cold water, and swelling up into a viscous mass when treated with hot water. Almost any drug may at will be incorporated with the bassorin base. If the one used be soluble in the water contained in the paste the colour of the latter is unchanged, but if insoluble, then the colour will vary according to that of the substance added to it. Those drugs which exist only as fluids cannot be mixed with the base, neither can alcoholic solutions; but fats may be, yet to a limited extent. The former are excluded for the reason that they render the base too liquid, while the alcohols cause it to become brittle and hard, and fats impair its property of drying and adhering to the skin. All substances, however, which exist in a solid form are easily incorporated in proportions as large as are ever used or found necessary. For instance, zinc oxide may be mixed with it to the extent of thirty or more per cent.; carbonate of magnesia, ten or more; chrysarobin, fifteen or more; salicylic acid, salicylate of soda, iodoform, resorcin, boric acid, borate of soda, sulphur, &c. Each and all incorporate perfectly with the bassorin paste without damaging its nature or its properties. Pixliquida, oleum rusci, and also icthyol, can be added successfully to the amount of fifteen per cent. The exact details of the preparation of bassorin paste are not given, but Dr. Elliott promises that Mr. Lascar will do so very shortly, when we shall communicate the information to our readers.

In conclusion, Dr. Elliott summarizes his observations as follows: (1) Bassorin paste is a perfectly neutral substance which, of itself, produces no irritation whatever, and when used alone it acts simply as a protective to the skin. It does not become rancid or decompose or undergo change when kept for a length of time, unless it be exposed in an open vessel. When this is done it becomes dry and hard, but even then rubbing it up with a little water renders it again as serviceable as at first. (2) It is easy and simple in application, requiring only to be spread upon the skin with the finger or a brush. It dries in the space of a few minutes if so applied, adheres closely, does not rub off and soil the linen, but forms a flexible coat, which does not interfere with the movements of the body. When its removal is desired, the preparation can be washed off with a little water, a damp cloth, or a sponge. It remains *in situ* without change for a variable length of time, depending upon the condition of the surface to which it has been applied. (3) With the bassorin paste almost anything can be incorporated; those which exist in the form of powders or in solid forms in any amount desired; the tars, icthyol, and oily substances in smaller percentages, but sufficient for all practical purposes. (4) The action of drugs incorporated with it and their effect upon disease appear to be as good as when such are used in other excipients, or perhaps better, in some cases. (5) It is of wide applicability, and of value in both acute and chronic forms of disease, its use being limited only by the degree of moisture on the surface being treated, or to which it may be exposed.

These properties, possessed by the bassorin paste, render it, as may be seen, superior to ointments, for the reasons, among others, that it is difficult to keep the latter in constant contact with the diseased surface, that salves soil and stain the linen, and offer such other objectionable features as greasiness, risk of being rancid, etc., to say nothing of the discomfort entailed in using them. It is also of wider application than the collodions. The bassorin paste would also seem to be superior to the gelatine preparations, in that these latter are more or less cumbersome in their application, having to be heated freshly whenever used.

